# Beyond Creatinine: D-Amino Acids as Emerging Indicators of Kidney Function and Disease

**DOI:** 10.3390/pharmaceutics18101207

**Published:** 2026-09-23

**Authors:** Alina Uifălean, Maria Iacobescu, Ximena Maria Mureșan, Simona Codruța Hegheş, Liana Claudia Salanță, Radu-Cristian Moldovan, Cristina-Adela Iuga

**Affiliations:** 1 Department of Pharmaceutical Analysis, Faculty of Pharmacy, “Iuliu Hațieganu” University of Medicine and Pharmacy, Louis Pasteur Street 6, 400349 Cluj-Napoca, Romania; alina.uifalean@umfcluj.ro (A.U.); cmaier@umfcluj.ro (S.C.H.); cristina.iuga@medfuture.ro (C.-A.I.); 2 Department of Personalized Medicine and Rare Diseases, MEDFUTURE—Institute for Biomedical Research, “Iuliu Hațieganu” University of Medicine and Pharmacy, Louis Pasteur Street 6, 400349 Cluj-Napoca, Romania; maria.iacobescu@medfuture.ro (M.I.); ximena.muresan@umfcluj.ro (X.M.M.); 3 Department of Food Science, Faculty of Food Science and Technology, University of Agricultural Sciences and Veterinary Medicine Cluj-Napoca, 400372 Cluj-Napoca, Romania; liana.salanta@usamvcluj.ro

**Keywords:** D-amino acids, chiral metabolomics, chronic kidney disease, D-serine, D-asparagine, gut–kidney axis, biomarkers

## Abstract

Despite decades of clinical use, serum creatinine and cystatin C remain imperfect indicators of kidney function, being influenced by muscle mass, age, sex, and inflammation and often showing limited sensitivity to early renal injury. D-amino acids (D-AAs), once largely regarded as by-products of microbial metabolism, have emerged as biologically active chiral metabolites with promising potential for improving the assessment of renal function and disease. This review synthesizes current mechanistic, analytical, and clinical evidence on D-AAs in chronic kidney disease, tracing their origins in diet, gut microbiota, and endogenous racemization through their stereoselective renal transport, tubular handling, and urinary excretion. Among these molecules, D-serine and D-asparagine have emerged as particularly promising biomarkers, showing strong associations with measured glomerular filtration rate and, in early studies, performance comparable or superior to conventional markers, though evidence remains limited to small or selected cohorts, while appearing less dependent on body composition across adult and pediatric populations. Beyond their biomarker potential, D-AAs participate in signaling and metabolic processes linking the gut, kidney, and brain, with altered D-AA homeostasis implicated in both renal injury and uremic neurological dysfunction. We critically examine the biological and clinical confounders, analytical challenges, and validation gaps that currently limit their translation into clinical practice and discuss how chiral metabolomics could contribute to a more precise assessment of kidney function and disease.

## 1. Introduction

In 2023, chronic kidney disease (CKD) was estimated to affect 788 million adults aged ≥20 years worldwide [1], ranking as the ninth leading cause of death globally, equivalent to one death every 20 s [2]. The rising global prevalence of obesity and diabetes, together with population aging, is expected to further amplify the overall burden of CKD [1]. The perspectives are grim, as by 2050, CKD is predicted to become the third leading cause of death in Western Europe [2].

CKD is recognized as a major global health issue, and an important driver of cardiovascular mortality and frailty. Its most severe clinical consequence is progression to end-stage kidney disease (ESKD), a condition in which patients require renal replacement therapy, including dialysis and/or kidney transplantation [3]. Therefore, early diagnosis and effective management are essential to identify high-risk patients and permit timely intervention to slow disease progression and improve patient outcomes.

Diagnosis and monitoring of CKD currently rely on biomarkers with well-documented limitations. Serum creatinine, widely used to estimate kidney function, is influenced by non-renal factors such as muscle mass, sex, age, and dietary protein intake, and it is often insensitive in detecting early kidney damage, particularly in patients with preserved renal function [4]. Cystatin C partially overcomes these limitations but is itself affected by systemic inflammation, adiposity, thyroid disease, and steroid use [5]. Inulin clearance, the gold standard for GFR measurement, requires continuous intravenous infusion and is analytically imprecise (coefficient of variation of approximately 7% for repeated measurements) and labor-intensive, which has limited its clinical use [6]. An ideal GFR biomarker should meet several key criteria: (i) free filtration across the glomerular membrane without protein binding, (ii) absence of significant tubular reabsorption or secretion, (iii) constant production rate, (iv) stable kinetics, independent of changes in GFR, and (v) accurate, easily accessible quantification in blood or urine [7]. In practice, no currently available biomarker fully satisfies all these conditions simultaneously, emphasizing the need for more precise indicators of kidney function.

Against this background, D-amino acids (D-AAs) have emerged as a distinct class of endogenous molecules, with promising potential as biomarkers of renal function. Long considered to be confined to bacterial cell wall peptidoglycans and certain invertebrate neuropeptides, D-AAs were first identified in mammalian brain tissue in the early 1990s [8] and were subsequently detected in human plasma, urine, cerebrospinal fluid, and saliva [9,10,11]. The cloning of serine racemase [12] established that D-serine (D-Ser) is synthesized endogenously in mammalian tissues, fundamentally revising the assumption that D-AAs are exclusively of microbial origin. Subsequent advances in chiral analytical chemistry enabled their systematic quantification in biological matrices, revealing that D-AAs are functionally active molecules with roles in neurotransmission, renal physiology, metabolic regulation, host–microbe interactions [13,14] and cancer-related processes [15,16]. The kidney occupies a central role in this biology by regulating the filtration, chiral-selective tubular handling, and metabolism of D-AAs, while simultaneously becoming vulnerable to their pathological accumulation in CKD [17,18].

In the context of CKD, D-AAs represent promising candidate biomarkers of renal function. Plasma concentrations of multiple D-AAs have shown strong correlations with established renal parameters, including blood urea nitrogen, serum creatinine, and cystatin C [19]. In particular, D-Ser [20] and D-Asparagine (D-Asn) [21] have been reported to closely reflect glomerular filtration rate (GFR) status with sensitivity and accuracy comparable or superior to conventional markers, while remaining largely independent of body size [22]. However, these findings derive mainly from relatively small or selected cohorts and warrant confirmation across broader CKD populations. Plasma D-Asn and the D/L-Ser ratio have also shown high sensitivity and specificity for detecting CKD in a pediatric cohort, independent of body size [23], pending validation in larger populations.

This review integrates current mechanistic, analytical, and clinical evidence to evaluate the role of D-AAs in CKD, critically appraising the biomarker evidence, identifying key mechanistic gaps, and framing chiral metabolomics within the broader vision of precision nephrology.

## 2. Sources of D-AAs: Diet, Microbiota, and Endogenous Production

D-AAs originate from three primary sources: dietary intake, microbial activity (specifically gut microbiota), and endogenous synthesis within mammalian tissues. Their relative abundance varies considerably depending on the dietary source, the type of biological fluid analyzed, and the pathological status of the organism [24,25].

### 2.1. Dietary Sources and Concentration-Dependent Effects

D-AAs are now recognized as being widely present in a variety of common food products and beverages, originating from natural sources, microbial activity, and industrial processing [24,25,26]. Fruits and vegetables contain low levels of D-AAs, in the range of about 0.5–3% relative to their L-enantiomers [27]. In contrast, marine invertebrates, such as oysters, mussels, scallops, and crustaceans, are among the richest natural sources, containing very high concentrations of D-Ala and D-Asp. In some bivalve mollusks, D-Ala can represent up to 90% of the total alanine content [25].

High concentrations of D-AAs are also found in fermented foods, such as cheese, yogurt, soy sauce, miso, and natto [24,26]. Fermentation serves as a “biological amplifier” of D-AAs, as bacteria and fungi produce these compounds as structural elements of their cell walls (peptidoglycan) or as secondary metabolites [26]. Growing evidence indicates that D-AA accumulation during fermentation is driven by strain-specific metabolic features, particularly racemase activity and amino acid transformation pathways, rather than the available energy source [26]. Analysis of 70 strains representing 17 *Lactobacillus* taxa demonstrated that amino acid concentrations were strongly associated with the phylogenetic grouping of *Lactobacillus*. Group-specific differences were primarily driven by variations in D-Ala and L-Ser concentrations. The *L. delbrueckii* group, often used as starters, was identified as a particularly high producer of D-Ala, which can influence the final taste profile of the product [28]. Similarly, D-AA profiles in alcoholic beverages are shaped by yeast and bacterial metabolism, as well as post-fermentation processing conditions. Wine aging is associated with progressive accumulation of D-Pro and other D-AAs, whereas beer and sake predominantly contain D-Ala, D-Glu, and D-Asp [26,29,30].

Beyond fermentation, heat-induced chemical racemization during food processing, particularly under alkaline conditions or at high temperatures, increases D-AA content in a variety of manufactured products, such as infant formulas, black ripe olives, sausages or coffee creamers [24].

An unprocessed or raw food diet is associated with a relatively low estimated total D-AA intake of approximately 10–30 mg/day, whereas a standard Western diet, characterized by high consumption of fermented and ultra-processed foods, may increase intake to an estimated 50–100 mg/day [24].

The wide variability in dietary D-AA intake is biologically relevant because the physiological effects of these compounds are strongly influenced by their concentration and the host context. Accordingly, Yamasaki et al. [26] described dietary D-AAs as “contronymic” molecules, highlighting their ability to exert opposing biological effects depending on dose, physiological context, and host metabolic state. The double-edged behavior of D-AAs reflects recurring mechanistic tradeoffs. At the biochemical core, D-amino acid oxidase (DAAO) degrades D-AAs into hydrogen peroxide, which at low levels functions as an adaptive “eustress” signal, supporting antimicrobial defense in the gut, but becomes a driver of oxidative “distress” and cell death when D-AA levels are excessive or antioxidant capacity is impaired [26]. This duality plays out across organ systems: in the kidney, physiological or low-dose D-Ser and D-Ala are renoprotective, promoting compensatory remodeling [18,31], whereas high-dose D-Ser acts as a uremic toxin causing mitochondria-dependent apoptosis and senescence [32,33]. In the brain, D-Ser and D-Asp serve as essential N-methyl-D-aspartate (NMDA) receptor co-agonists, enabling synaptic plasticity and memory. However, their overabundance, especially extrasynaptically, triggers excitotoxicity linked to neurodegeneration [26,34]. Underlying all of this is host vulnerability: in healthy individuals, D-AAs support innate defense, but in the elderly or those with CKD, impaired clearance allows them to accumulate, converting useful signals into stressors that amplify inflammation and oxidative burden [13,26]. D-Ser dose-dependent mechanisms are further detailed in Section 5.1.

### 2.2. Gut Microbiota as the Dominant Contributor

The intestinal microbiota is recognized as a major source of D-AAs in mammals [35], contributing substantially to the overall human D-AA burden, though the precise dynamics of this contribution remain inconclusive [13]. A stable isotope tracer study revealed that D-Ala levels were significantly higher in conventional mice compared to germ-free mice, particularly in the colon, confirming the microbiome’s role as a major source [36].

Further evidence comes from intervention studies, in which antibiotic treatment with amoxicillin reduced urinary D-Ala levels by approximately six- to eight-fold compared with untreated controls [37], while germ-free mice showed markedly diminished urinary D-Ala concentrations despite normal dietary intake, confirming that intestinal bacteria rather than diet per se are the primary source of this specific D-AA [38]. Bacterial D-AA synthesis occurs via two principal enzymatic routes: amino acid racemases and epimerases, which catalyze the direct interconversion of L- and D-stereoisomers through a pyridoxal phosphate-dependent mechanism, and D-AA transaminases, which generate D-AAs through transamination of α-keto acids using D-glutamate as the amino donor [39,40]. Different bacterial species possess racemases with varying substrate specificities. Alanine racemase is essentially ubiquitous in bacteria and the primary source of D-Ala incorporated into peptidoglycan [39]. Beyond Ala, diverse bacteria, ranging from *Vibrio cholerae* and *Aeromonas hydrophila* to *Bacillus subtilis* and *Pseudomonas putida*, utilize both broad-spectrum and specific racemases to process an extensive array of substrates, including Glu, Ser, Asp, Pro, and Lys, to produce non-canonical D-AAs essential for cell wall modulation and microbial physiology [41,42,43,44]. The activity of these diverse racemases generates a unique, individual-specific profile of D-AAs. They act as inter-domain signaling molecules that can affect host processes, such as immune responses and even brain function through the gut–brain axis [24].

The relevance of gut microbiota as a D-AA source acquires particular importance in CKD, where intestinal dysbiosis is a well-documented and functionally significant complication [45,46]. CKD-associated dysbiosis is characterized by a progressive shift from a healthy, carbohydrate-fermenting state to a protein-fermenting one, marked by overgrowth of pathogenic bacteria (especially *Proteobacteria* and *Enterobacteriaceae*) and depletion of beneficial commensals like *Lactobacillus* and *Bifidobacterium* [47,48]. This dysbiosis expands uremic toxin-producing enzymes (urease, tryptophanase) while reducing short-chain fatty acid-producing bacteria [49], compromising gut barrier integrity and fueling systemic inflammation through lipopolysaccharide translocation and accumulation of toxins such as indoxyl sulfate, p-cresyl sulfate, and trimethylamine N-oxide [49].

These compositional changes alter the microbial enzymatic capacity for D-AA synthesis, reducing production of potentially protective D-AAs, while potentially altering the balance of D-AA substrates available for downstream metabolism. The implication is that CKD dysbiosis creates a self-amplifying cycle: impaired renal function alters the gut microbiome, which in turn modifies the D-AA milieu, which feeds back on both renal tubular biology and systemic inflammation [26,50]. A detailed discussion of these mechanisms is provided in Section 5.

### 2.3. Endogenous Production

Endogenous D-AA synthesis in mammalian tissues occurs principally through enzymatic racemization. Serine racemase, cloned in 1999 by Wolosker et al. [12,51], catalyzes the reversible conversion of L-Ser to D-Ser through a pyridoxal phosphate-dependent α,β-elimination and re-addition mechanism. It is also capable of direct serine dehydration to pyruvate and ammonia under certain conditions [52]. The detection of serine racemase in renal cells has raised the possibility of local D-Ser synthesis within the kidney itself, independent of glomerular filtration [24].

Enzymatic degradation of D-AAs is mediated primarily by DAAO (EC 1.4.3.3), a peroxisomal flavoprotein that catalyzes the oxidative deamination of neutral and basic D-AAs to yield the corresponding α-keto acids, ammonia, and hydrogen peroxide. D-aspartate oxidase performs an analogous function for acidic D-AAs including D-Asp and D-Glu [50].

While diet and gut microbiota are major contributors to the D-AA pool, spontaneous racemization serves as a distinct endogenous source, governed by thermodynamic rather than catalytic principles [26]. This source gains pathological relevance in the context of renal decline. Spontaneous racemization occurs at a slow but constant rate in long-lived proteins located in metabolically stable or avascular tissues [13]. The accumulation of these “wrong” enantiomers leads to the denaturation of essential proteins, which is suggested as a contributing factor to the age-related decline in organ function, including the kidneys [24].

### 2.4. Critical Gap

Despite the multiplicity of D-AA sources identified, the quantitative contribution of each pathway to the circulating and urinary D-AA pools in humans remains unclear, both under physiological conditions and across different stages of CKD. Most studies rely on static metabolite concentrations, which fail to capture the dynamic flux of D-AAs through renal and systemic pathways. Clinical in vivo flux studies using isotope tracers are currently lacking in humans to partition microbial, dietary, and endogenous contributions [53]. Inter-individual variability in microbiome composition, dietary habits, DAAO activity, serine racemase expression, and renal transporter profiles creates a confounding landscape that observational metabolomic studies cannot resolve. Notably, sex differences in these parameters, including known sexual dimorphism of renal transporters and potentially of DAAO expression, are entirely unstudied in the context of D-AA metabolism [53]. Moreover, quantitative thresholds defining a transition from “physiological” to “pathological” D-AA levels have not been established [13,50]. Finally, alterations in D-AAs (e.g., elevated D-Ser) are observed across multiple unrelated conditions, including acute kidney injury (AKI), CKD, and various neurological disorders, raising concerns regarding their diagnostic specificity [50].

## 3. Transport and Renal Handling of D-AAs

The transport of D-AAs is mediated by specialized systems with distinct substrate specificities and organ localizations. While most amino acid transporters have a much higher affinity for L-enantiomers, several families can handle also D-AAs, playing important roles in their intestinal absorption, renal reabsorption, and tissue-specific uptake, including in the kidneys.

The integrated function of the kidney in D-AA homeostasis extends beyond transporter-mediated reabsorption and rests on several complementary mechanisms including DAAO-mediated enzymatic degradation and urinary excretion. The relative contribution of each mechanism is not uniform among individual D-AA species and is not constant across disease states, a distinction with important implications for interpreting plasma D-AA accumulation in pathological contexts.

This chapter traces the trajectory of D-AAs from dietary and microbial sources through to intestinal absorption, renal filtration and selective reabsorption, enzymatic degradation, and ultimately urinary excretion.

### 3.1. Intestinal Absorption Systems

Absorption from diet and gut microbiota occurs primarily in the small and large intestines, where several apical transporters mediate their entry into epithelial cells. The proton-coupled transporter hPAT1 (SLC36A1) represents a major entry point for small neutral D-AAs in the small intestine. It exhibits a 6–8-fold higher affinity for D-Ser and D-Cys than for their corresponding L-enantiomers, whereas similar affinities are observed for the L- and D-forms of Ala and Pro [24,54]. ATB^0,+^, expressed in the distal small intestine and colon, is a sodium-dependent transporter with high affinity for D-Ser, D-Ala, D-Met, D-Leu and D-Tyr, making it uniquely suited for the absorption of bacterially produced D-AAs into the circulation [55]. The literature also mentions PEPT1 (SLC15A1), an intestinal and renal proton-coupled symporter that primarily transports dipeptides and tripeptides. PEPT1 may contribute to the intestinal uptake of small peptides containing D-AA residues, although transport efficiency generally depends on peptide structure and stereochemical configuration [56].

Once absorbed across the apical membrane, D-AAs must cross the basolateral membrane to enter the portal circulation. Facilitative transporters, such as LAT1 (SLC7A5), ASCT1 (SLC1A4), and ASCT2 (SLC1A5), contribute to amino acid homeostasis and regulate the systemic distribution of D-AAs, including their selective transport across the blood–brain barrier [55,57,58]. These basolateral systems thus serve as the functional bridge between intestinal uptake and systemic bioavailability, and their substrate preferences ultimately determine which D-AA species reach the kidney in sufficient concentrations to be subject to renal regulation [24,35,50]. 

### 3.2. Renal Reabsorption and Chiral Selectivity

The kidney acts as the principal regulator of systemic D-AA levels [18,20,35]. The tubular reabsorption of D-AAs is governed by a sequential interplay between transmembrane transport and intracellular metabolism.

In the early proximal tubule (S1 and S2 segments), the major apical reclamation systems, including B0AT1 (SLC6A19), rBAT/SLC3A1, and related SLC7 family exchangers, exhibit markedly lower affinity for D-enantiomers than for their corresponding L-counterparts. Therefore, L-AAs are extensively reabsorbed (up to 99%), while D-AAs escape reabsorption and become progressively concentrated in the tubular fluid as it advances toward the S3 segment of the proximal tubule [24].

The S3 segment (pars recta) is the key site for active D-AA reabsorption. Here, apical transporters mediate the uptake of filtered D-AAs from the tubular lumen into proximal tubular epithelial cells. Once inside the cell, D-AAs encounter an important intracellular metabolic barrier represented by DAAO. Localized in peroxisomes, DAAO oxidatively deaminates a substantial fraction of reabsorbed D-AAs, generating the corresponding α-keto acids, ammonia, and hydrogen peroxide (H_2_O_2_), thereby limiting their return to the systemic circulation. Tubular reabsorption is therefore completed only for the fraction of D-AAs that escape enzymatic degradation, whose efflux across the basolateral membrane into the peritubular circulation is mediated by basolateral transporters [59].

From the tubular fluid, ATB^0,+^ (SLC6A14) mediates the uptake of D-Ala, D-Met, D-Leu, and D-Trp, while exhibiting comparable affinity for both L-Ser and D-Ser [55]. In parallel, EAAT3 (SLC1A1), the only anionic amino acid transporter at the apical membrane, displays relatively high stereoselectivity for D-Asp [24]. PAT2 (SLC36A2), a proton-amino acid symporter, prefers L- over D-Ala, but transports L- and D-Ser with equal affinity [60]. An additional contribution is provided by SMCT1 and SMCT2 (SLC5A8/12), sodium-coupled monocarboxylate transporters that reabsorb D-Ser as a “hidden” or non-canonical substrate [18,61]. Apical reabsorption of D-Ser can also be accomplished through ASCT2 (SLC1A5) [62].

Basolateral transport (efflux to blood) is mediated by Asc-1 (SLC7A10), an antiporter with high affinity for D-Ser, D-Ala, and D-Cys that exchanges them for L-AAs [24,62], and LAT4 (SLC43A2), a uniporter facilitating the efflux of D-Leu, D-Phe, D-Val, D-Met and their corresponding L-forms [63].

An in-depth description of D-AA transport systems is presented in the work of Roskjær et al. [24] and Knol et al. [63].

The stereoselective behavior of D-AA transport is influenced by both physiological factors and disease-induced alterations. Under physiological conditions, competition with the corresponding L-AAs represents a major determinant, as D-AAs often share transport systems with their far more abundant L-counterparts, limiting their access to carrier binding sites [24]. Transport activity is also modulated by the physicochemical environment, with certain systems, such as PAT1 and PEPT1, relying on proton gradients, whereas others, including ATB^0,+^, depend on sodium and chloride gradients [25]. In pathological states, renal injury can profoundly disrupt stereoselective handling by altering transporter function and expression.

The consequences of this chiral selectivity in pathological states are precisely described in the landmark study by Hesaka et al. [20], which measured fractional excretion (FE) of chiral amino acids alongside inulin clearance using two-dimensional HPLC with fluorescence detection. In non-CKD subjects, the FE of D-Ser was 62.1% [53.4–73.6%] and the FE of D-Ala was 20.7% [17.7–22.2%], contrasting sharply with the FE of L-amino acids, which uniformly remained below 2.5%. The %D ratio, defined as D/(D + L) × 100, of Ser in non-CKD urine reached 45.4%, compared with 1.46% in plasma, an approximately 30-fold enrichment of the D-form in urine that directly reflects the chiral selectivity of tubular reabsorption. These data confirm that the healthy kidney maintains low plasma D-AA concentrations through continuous preferential urinary elimination. When GFR declines, reduced tubular delivery leads to progressive plasma accumulation proportional to the degree of filtration loss. Tanaka et al. [64] formalized this principle by demonstrating that both D-Ser and D-Asn exhibit FE patterns closely approximating those of inulin, consistent with near-complete filtration and minimal net tubular transport [21]. This observation underlies their proposed utility as endogenous GFR markers.

### 3.3. DAAO: Central Metabolic Enzyme and Its Vulnerability in CKD

D-amino acid oxidase (DAAO) is expressed predominantly in the peroxisomes of proximal tubular cells, with the highest enzymatic activity in the S3 segment (pars recta) [65], and to a lesser extent in the intestinal epithelium and cerebellum. Its catalytic activity generates H_2_O_2_ as a stoichiometric by-product of D-AA oxidation [66]. In the proximal tubule context, this local H_2_O_2_ production may contribute to host defense against urinary tract pathogens, a physiologically underappreciated function that positions DAAO at the interface of amino acid metabolism and innate immunity [35,67].

An important mechanistic distinction, emphasized by Tanaka et al. [64], is that under normal physiological conditions the dominant mechanism maintaining low plasma D-AA concentrations is renal excretion, filtration-dependent, rather than DAAO-mediated catabolism. D-Ser, for instance, is almost entirely eliminated via urine rather than oxidized intracellularly, which explains why its FE approximates that of inulin [21]. This distinction carries a direct implication for interpreting plasma D-AA accumulation in CKD: the primary driver is reduced GFR and hence diminished tubular delivery and excretion, not a decrease in DAAO enzymatic activity per se.

However, DAAO inactivation acquires pathological significance specifically in the context of acute tubular injury. Ischemia–reperfusion injury inactivates DAAO in proximal tubular cells, leading to acute accumulation of D-Ser and perturbation of Ser enantiomer homeostasis in both plasma and urine. In ischemia–reperfusion injury mouse models, the L/D-Ser ratio in blood and urine provided earlier and more sensitive detection of renal injury than established markers including KIM-1, NGAL, creatinine, cystatin C, and urea, positioning Ser chirality as a real-time reporter of tubular oxidative and ischemic stress [35,68]. This distinguishes D-Ser’s role as an acute-injury marker from its use in GFR estimation, discussed separately in Section 4.1.

### 3.4. Renal Excretion of D-AAs

While L-amino acids are conserved for protein synthesis, the body treats D-AAs as bioactive signaling molecules or metabolic by-products, primarily managing their levels through filtration-dependent urinary elimination.

Landmark studies in healthy human subjects have established characteristic urinary excretion patterns for key D-enantiomers. These patterns are typically expressed as FE, defined as the ratio of the clearance of a given substance to the clearance of a standard reference molecule. D-Asn shows a median FE of approximately 98.7%, closely approximating inulin clearance and indicating minimal net tubular transport [21,53]. D-Ser exhibits an FE of around 62.1% (up to ~85% when inulin-corrected), reflecting substantial urinary loss with reabsorption that is highly sensitive to renal injury [20,21]. In contrast, D-Ala shows a lower FE of approximately 20.7%, consistent with significant tubular reabsorption and reported circadian variation [14,20]. D-Pro is almost completely retained, with an FE of ~0.1%, reflecting highly efficient reabsorption and/or metabolism, resulting in only trace urinary levels under physiological conditions [19].

The physiological journey of D-AAs, from their origins in the gut microbiota and dietary intake through to intestinal epithelial transport and subsequent renal handling, is illustrated in Figure 1.

### 3.5. Disruptions in Kidney Disease

Kidney disease profoundly disrupts the transport dynamics described above, contributing to the systemic accumulation of D-AAs through several mechanisms. First, direct tubular damage impairs reabsorptive capacity: in diabetic nephropathy, proximal tubule injury leads to a significant increase in the FE of D-Ser [69], reflecting both transporter dysfunction and altered tubular fluid dynamics. Second, alterations can occur also in transporter induction. It was shown that proximal renal tubular damage (e.g., from cisplatin) can induce the expression of Asc-1 in tubules and collecting ducts, which was associated with elevated blood levels of D-Ser [70]. In particular, because D-AAs share transporters with L-AAs, their reabsorption is normally limited by competition with high L-AA levels [24,71]. This competitive balance is frequently disrupted in disease states, contributing to aberrant excretion and plasma accumulation patterns that may serve as sensitive indicators of tubular dysfunction.

## 4. D-AAs as Emerging Biomarkers in CKD

Traditional indicators of CKD, including serum creatinine and estimated glomerular filtration rate (eGFR), lack sensitivity for detecting early kidney damage, often remaining within the normal reference range until approximately 50% of renal function has already been lost [72,73]. In addition, these markers are substantially influenced by extra-renal factors such as muscle mass, age, sex, and dietary intake, while proteinuria exhibits considerable biological variability [73]. Combined with the largely asymptomatic progression of CKD, these limitations frequently delay diagnosis until irreversible nephron loss has occurred. Therefore, there is an urgent need for novel, more sensitive, and more specific biomarkers that enable earlier detection, improve risk stratification, and facilitate timely therapeutic intervention [73,74].

Table 1 summarizes the most representative emerging biomarkers for CKD, highlighting their clinical applications, advantages, limitations, and current validation status. A more comprehensive overview of emerging biomarkers in CKD is available in recent dedicated reviews [72,73,74]. 

While these emerging biomarkers have substantially improved the characterization of renal injury and CKD progression, they primarily reflect structural damage, tubular stress, or alterations in filtration. In contrast, D-AAs represent an emerging class of metabolic biomarkers that may provide complementary insights into CKD pathophysiology by capturing alterations in amino acid metabolism and renal handling.

The accumulation of D-AA in CKD follows a stage-dependent pattern, primarily reflecting the progressive decline in glomerular filtration rather than increased endogenous production or reduced enzymatic degradation. In the study of Kimura et al. [82], plasma samples from 108 CKD patients were analyzed by two-dimensional high-performance liquid chromatography (2D-HPLC), revealing that D-Ser, D-Asn, and D-Pro clustered with conventional renal markers, including creatinine and cystatin C, confirming that D-enantiomer accumulation is governed by GFR-dependent renal impairment rather than amino acid identity per se. Subsequent quantitative analyses demonstrated that both absolute plasma concentrations and %D ratios increase progressively with declining eGFR for a broad panel of D-AAs, including D-Ser, D-Ala, D-Pro, D-Glu, D-Lys, D-Leu, and D-Phe [13] (Table 2). The data consistently demonstrate that D-Ser and D-Asn are more accurate endogenous markers for eGFR than creatinine and can be used to estimate urine volume [83]. The mechanistic basis for this gradation has been formalized by Tanaka et al. [64], who established that the primary driver of plasma D-AA accumulation is reduced glomerular delivery and tubular excretion, not increased racemase activity or decreased DAAO function. This distinction is important for biomarker interpretation, as D-AAs, particularly D-Ser and D-Asn, may provide a more accurate reflection of declining kidney filtration function than creatinine, whose tubular secretion can result in eGFR overestimation.

So far, only D-Ser and D-Asn have been validated against inulin clearance, the physiological gold standard for GFR assessment. Therefore, the following discussion focuses on these two D-amino acids as the best-validated D-AA markers of kidney function.

**Table 2:** D-AAs as biomarkers in CKD: diagnostic accuracy, prognostic utility, and clinical context across disease stages and populations.

Category/ Utility	Study Design; n = Sample Size (n CKD; n Controls)	Sample Type	eGFR_creat_/Stage	D-AAs Measured	Main Observations	Ref.
**Precision GFR sensing**	Cross-sectional retrospective; n = 26 (11; 15)	Plasma and urine	79.3–93.5 (non-CKD)	Plasma µM: D-Ser 1.56 (1.15–1.87); D-Ala: 0.99 (0.71–1.82); D-Pro: 0.48 (0.33–0.62). FE: D-Ser 62.1%, D-Ala 20.7%.	▪ Plasma D-Ser levels correlate strongly with actual GFR (inulin clearance) and increase in CKD independently of body size or sex; ▪ Combined plasma level and FE of D-Ser distinguish CKD from controls with 72.7% sensitivity and 100% specificity.	[20]
			21.7–89.2 (CKD)	Plasma µM: D-Ser 2.39 (1.55–6.08), D-Ala 1.28 (0.74–2.86), D-Pro 0.78 (0.50–1.57); FE: D-Ser 50.5%, D-Ala 22.8%.		
	Cross-sectional; n = 207 (129 donors; 49 recipients; 29 post-donors)	Plasma and serum	68.22 ± 19.49	Plasma µM: D-Ser: 2.21 ± 0.78; D-Asn: 0.21 ± 0.10; D-Ala: 2.26 ± 2.2; D-Pro: 0.88 ± 0.77. FE: D-Asn 98.7%, D-Ser 84.8%	▪ D-Asn clearance was almost identical to inulin (FE~98.7%); it presents minimal proportional bias (−7.8%), eliminating the creatinine-specific GFR overestimation caused by tubular secretion.	[21]
**Early CKD detection**	Cross-sectional; n = 180 (165; 15)	Plasma	5.5–104.1 (pan-stadial)	µM: D-Ser 0.95–19.0; D-Thr 0–0.57; D-aThr 0.04–1.02. %D: Ser 0.99–19.0%; Thr 0–0.72%; aThr 0.04–1.94%.	▪ Thr enantiomers (D-Thr and D-aThr) increase progressively with decreasing GFR, serving as supplementary biomarkers to enhance diagnostic accuracy in multi-analyte panels.	[84]
	Cross-sectional; n = 20 (15; 5)	Plasma	33.1–67.2 (Stage 2/3)	μM: D-Ser 2.45–10.02; D-Ala 1.99–7.22; D-Pro 1.15–2.56; D-Lys 0.14–0.37; D-Phe 0.04–0.09.	▪ D-Ala, D-Pro, and D-Ser increased stage-dependently, while %D-Ser and %D-Phe showed strong correlations with eGFR. ▪ **Early-stage sensitivity:** D-Phe is a highly sensitive indicator of incipient renal dysfunction, accumulating in plasma before significant increases in sCr. ▪ Stage 5 patients showed the highest concentrations and %D values of all measured D-AAs, including D-Glu.	[85]
			18.2–25.4 (Stage 4)	μM: D-Ser 4.20–10.48, D-Ala 5.82–17.34, D-Pro 0.60–11.63, D-Lys 0.28–4.34, D-Phe 0.05–0.09, D-Leu 0.12–0.22.		
			9.4–13.4 (Stage 5)	μM: D-Ser 8.50–11.12, D-Ala 10.64–24.66, D-Pro 2.05–7.38, D-Lys 0.43–0.83, D-Phe 0.07–0.13, D-Glu 0.09–0.50, D-Leu 0.14–0.38.		
**Prognostic prediction**	Longitudinal observational cohort; n = 108 (108; 0)	Plasma	21.0 ± 12.4 (Stages 3–5, not on dialysis)	16/21 D-AAs detected (absolute values not reported).	▪ D-Ser, D-Pro, and D-Asn were inversely associated with eGFR. ▪ **Prognostic power:** Higher plasma D-Ser and D-Asn levels predicted a 2.7–3.8-fold greater risk of ESKD or death before ESKD.	[82]
	Cross-sectional and prospective; n = 65 (30 CKD; 35)	Urine	65.3 ± 30.6	D-Ser 77.1 (59.0 ± 92.5) (nmol/mg creatinine). D/L-Ser ratio 0.65 (0.34 ± 1.06).	▪ **Deterioration risk:** Low urinary D-Ser (<69.8 nmol/mg creatinine) is associated with future eGFR decline and poor event-free survival → predictive biomarker for renal deterioration in patients with atherosclerotic risk factors.	[86]
**Longitudinal monitoring**	Longitudinal/Observational Pilot; n = 150 (135 DKD; 15 controls)	Serum and urine	47.8 [41.6–53.4]	Serum µM: D-Ser ~3.5; D-Ala ~6.2; D-Pro: ~2.7; Ratio: D/L-Ser ~2.8%.	▪ **Longitudinal predictor:** Short-term changes in D-Ser levels and the D/L-Ser ratio independently predict rapid eGFR decline (>10%/year) in diabetic patients, outperforming static measurements.	[87]
**Pediatric assessment**	Prospective; n = 27 (12 pediatric CKD; 15)	Plasma and urine	≥90	Plasma μM: D-Ser: 1.84 ± 0.31, D-Asn: 0.118 ± 0.027; Plasma D/L-Ser ratio 1.35 ± 0.16%, D/L-Asn ratio: 0.27 ± 0.07%.	▪ **Developmental stability:** Plasma D-Asn and the D/L-Ser ratio accurately reflect CKD stage independently of muscle mass or sex, addressing the major physiological limitations of sCr in pediatric practice.	[23]
			<90	Plasma μM: D-Ser 2.29 ± 0.70; D-Asn 0.187 ± 0.080; Plasma D/L-Ser ratio: 2.21 ± 0.7%, D/L-Asn ratio: 0.41 ± 0.11%.		
**IgAN management**	Observational cohort; n = 38 (17 IgAN; 21)	Urine	Stages 1–3 (exclusion of stages 4–5)	12 D-AAs identified, numeric values not reported.	▪ **Etiology-specific management:** Decreased urinary D-AAs (D-Ser, D-Asp, D-Thr) correlated with serum albumin and neutrophil counts, providing a non-invasive marker of IgA nephropathy activity.	[88]

Note: Values are reported as presented in the original studies (mean ± SD, median [IQR], or range, as applicable).

### 4.1. D-Serine as a Dual Biomarker: GFR Estimation and CKD Detection

Evidence identifies plasma D-Ser as a robust endogenous marker of GFR due to its strict renal regulation. Hesaka et al. [20] provided a rigorously designed study of D-Ser as a CKD biomarker, enrolling subjects who underwent simultaneous inulin clearance measurement (gold standard GFR) and comprehensive chiral amino acid metabolomics. Plasma D-Ser correlated with inulin-measured GFR with Kendall’s tau of r = −0.483 (*p* = 0.0006), a magnitude comparable to the correlations for cystatin C (r = −0.483) and superior to creatinine (r = −0.406, *p* = 0.0039). D-Ser clearance further showed lower proportional bias than creatinine clearance (22.0% vs. 34.6%) in a cohort of 200 kidney transplant donors and recipients [89]. Unlike creatinine, D-Ser is minimally influenced by muscle mass, sex, or body surface area [20], and its partial tubular reabsorption (~15–40%) remains stable across GFR ranges, contrasting with creatinine, whose tubular secretion introduces systematic variability [89]. D-Ser thus offers a potentially more accurate reflection of GFR in elderly patients with sarcopenia, in patients with obesity or edema, in pediatric patients where creatinine reference ranges are complicated by growth-dependent muscle mass changes, and in populations where ethnicity-specific creatinine equations are not validated [18,20,23,89].

Beyond GFR estimation, the combination of plasma D-Ser concentration with its FE achieved sensitivity of 72.7% and specificity of 100% for CKD detection when the 95% confidence ellipse of the non-CKD population was used as the classification boundary, a bivariate discriminant performance that neither plasma concentration nor FE alone achieved [20]. This dual-parameter approach exploits the fact that the two variables carry complementary pathophysiological information: plasma concentration reports on filtration adequacy, while FE reports on tubular reabsorptive integrity, and their combination provides a more complete picture of nephron function than any single variable. The underlying mechanism is related to the differential tubular reabsorption of D-Ser. While most L-AAs undergo nearly complete reabsorption, D-Ser reabsorption is particularly susceptible to alterations caused by renal injury. Consequently, its FE is tightly regulated in healthy kidneys (around 62%), but becomes highly variable or distinctly elevated under pathological conditions, including diabetic nephropathy [20].

### 4.2. D-Asparagine as an Endogenous GFR Measurement Tool

D-Asn has recently emerged as a highly promising endogenous biomarker for the measurement of GFR, with several studies identifying it as the most “inulin-like” endogenous molecule discovered to date [21,23,64]. Like exogenous inulin, it is a small molecule (132.12 g/mol molecular weight) that is freely filtered at the glomerulus and exhibits negligible tubular reabsorption or secretion [64].

Blood levels of D-Asn and the D/L-Asn ratio show a strong inverse correlation with inulin clearance. Taniguchi et al. [21] reported a Pearson’s correlation coefficient of *r* = −0.64 for blood D-Asn levels and R = 0.88 for D-Asn clearance against inulin clearance. This performance is supported by an FE of 98.67% (95% CI), indicating that D-Asn is handled almost exclusively by glomerular filtration, with minimal tubular involvement. As a result, D-Asn clearance avoids the systematic overestimation seen with creatinine clearance, which is influenced by tubular secretion, and shows less bias than D-Ser, which is partially reabsorbed in the proximal tubules and therefore exhibits greater variability [21].

Unlike creatinine, which is a product of muscle metabolism, D-Asn levels are not affected by body size, age, height, or weight. This makes it particularly valuable for pediatric populations where muscle mass changes rapidly during growth. Also, animal studies indicate that D-Asn is not influenced by glucocorticoids (steroids), which often cause false elevations in cystatin C levels [23].

Reduced urinary D-Asn has also been observed in the presence of glioblastoma, suggesting a potential for oncological overlap to confound its use as a purely renal marker in specific cases [90]. Pending broader validation, D-Asn is among the most promising endogenous candidates for approximating inulin clearance.

Beyond the direct validation against inulin clearance discussed above, D-Ser and D-Asn have undergone further cross-sectional and longitudinal validation in independent cohorts. Baseline levels of both markers independently predicted a 2.7–3.8-fold higher risk of progression to ESKD or death in a longitudinal cohort [82], while short-term fluctuations in blood D-Ser predicted rapid eGFR decline in DKD [87]. Incorporating D-Ser into a multivariate panel alongside classic risk factors also improved discrimination for CKD and CKD-to-ESKD progression, although without an independent external validation cohort [91]. In pediatric CKD, D-Asn and the D/L-Ser ratio have shown high diagnostic accuracy independent of body size [23]. Broader clinical translation will require harmonized protocols and large, multiethnic multicenter validation studies accounting for confounders such as diet, microbiome, and diurnal variation, several of which are examined in the following section.

### 4.3. Specificity and Confounding Factors in D-AA-Based CKD Biomarkers

The specificity of D-AAs as biomarkers in CKD remains a critical consideration, as their levels reflect not only renal dysfunction but also a range of disease-related, physiological, and analytical influences that limit absolute disease specificity [50].

Circulating levels of eGFR-correlating markers such as D-Ser are frequently altered in neuropathological comorbidities (e.g., schizophrenia, Alzheimer’s disease, and stroke) [92,93] due to their physiological role as an NMDA receptor co-agonists, while urinary D-Asn depletion is confounded by oncological conditions like glioblastoma [90] and bladder cancer [94]. Within the renal system itself, circulating D-AAs accumulate during AKI, as shown in both human and animal studies [31,95,96], and their intra-body clearance dynamics vary dramatically depending on the underlying primary kidney pathology, as demonstrated by the contrasting “plasma-high, FE-high” profile in diabetic nephropathy versus the “plasma-low, FE-low” profile in lupus nephritis [69,97].

Exogenous influences also introduce significant systemic baseline noise. Dietary intake of fermented or processed foods leads to high exposure and thermally induced racemization supported by food-chemistry and mechanistic data [24,98], while the symbiotic gut microbiota [99] serves as a primary generator of D-AAs. Crucially, elevations in circulating D-Ala in CKD patients with diabetes are heavily driven by oral *Streptococcus* species rather than eGFR clearance alone [100]. To circumvent the dietary confounding associated with D-Ser, structural analogs with lower dietary abundance, such as D-threonine and D-allo-threonine, have emerged, in human observational clinical data, as promising, diet-independent biomarker candidates [84].

In contrast to conventional, muscle mass-dependent markers like creatinine, D-Ser and D-Asn remain largely independent of body mass, age, and sex in GFR estimation models [22], offering a highly stable alternative for diverse patient populations. In both adult and pediatric cohorts, circulating levels of these D-AAs reflect renal clearance irrespective of body surface area, avoiding diagnostic inaccuracies in sarcopenic patients or during rapid pediatric growth phases [20,23]. Age-related impacts are minimal: while plasma D-Asn remains completely age-independent during development, the pediatric plasma D/L-Ser ratio displays a slight positive correlation with age. Baseline sex differences are also enantiomer-specific: plasma concentrations and D/L-ratios of D-Asn, D-Ala, and D-Pro are significantly higher in males than in females, whereas circulating D-Ser exhibits no significant sexual dimorphism [19]. Additionally, D-AA levels may exhibit pronounced circadian and diurnal fluctuations, as reported in animal models for D-Ala, with their excretion patterns influenced by sleep–wake and resting states [101].

Finally, because D-AAs exist in trace quantities (typically <1% to 3% of L-counterparts in blood) [85,87], precise quantification is hindered by analytical challenges, including interfering peaks in complex biological matrices, matrix effects on ionization, and requirements for absolute optical purity of chiral derivatizing agents to avoid quantitative bias [34,85,102].

### 4.4. Multimarker Panels

Single markers frequently fail because they are influenced by non-renal factors or only reflect one aspect of kidney function. In contrast, combined biomarker panels provide a more comprehensive assessment of nephron physiology. Blood-based markers (e.g., D-Ser, creatinine) primarily reflect GFR as systemic “sensors,” whereas urinary profiles and FE provide complementary information on tubular reabsorptive function and renal pathology [17,18,20].

Combining creatinine clearance, affected by tubular secretion, with D-Ser clearance, subject to partial reabsorption, mitigates their respective directional biases and improves GFR estimation accuracy beyond either marker alone. This is evidenced by P30 and P15 accuracies of 98.5% and 89.7%, respectively, versus the gold standard [89]. Plasma D-Ser and its FE were used by Hesaka et al. [20] to construct a 95% confidence ellipse based on a non-CKD reference population. This method identified CKD with a sensitivity of 72.7% and a specificity of 100%. Beyond GFR estimation and CKD classification, panels have also been evaluated for a distinct clinical task, predicting disease progression. A metabolic panel comprising D-Ser, citrulline, and the kynurenine/tryptophan ratio was shown to discriminate CKD stages and predict disease progression. When incorporated alongside conventional risk factors, including age, sex, body mass index, and diabetes status, this metabolic signature increased the AUC for CKD stage classification from 86.7% to 100% and enhanced the AUC for progression risk prediction from 63.0% to 96.5% [91]. The combined assessment of D-Ser with its structural analogs, D-threonine and D-allo-threonine, has been proposed to minimize interference arising from non-renal conditions [84].

Beyond correlation with measured GFR, D-Ser/D-Asn may offer particular clinical value in settings where creatinine-based eGFR is least reliable: sarcopenic patients [20], pediatric growth phases [23], and kidney donor/recipient evaluation [89]. Their clinical utility in these specific contexts, however, remains to be formally validated.

Several challenges currently limit the widespread clinical implementation of multimarker panels based on D-AAs. First, their analysis relies on specialized techniques such as 2D-HPLC, 3D-HPLC or LC–MS/MS, often requiring complex and time-consuming derivatization procedures [31,34,84,88,103]. Second, the lack of standardized analytical protocols highlights the need for interlaboratory harmonization and the development of automated, high-throughput platforms suitable for routine clinical use [26]. Finally, biological variability represents an additional challenge, as factors such as gut microbiota composition and dietary intake, particularly fermented foods, can substantially influence circulating levels of specific D-AAs and potentially affect panel performance.

### 4.5. Analytical Challenges/Methodological Considerations

Despite significant recent advances in the chiral analysis of D-AAs, only a handful of analytical approaches have been applied to the study of D-AAs associated with CKD (Table 3). Among these, the most representative are the 2D-LC-FL approaches. These are based on sample derivatization with a non-chiral derivatizing agent (typically NBD-F); then the derivatized analytes get separated on a reversed-phase platform in the first dimension, followed by chiral separation of the target enantiomers in the second dimension. This provides excellent selectivity and high detection sensitivity, offered by the fluorescence detection. This method was used for analysis of both plasma and urine of CKD patients, reporting absolute concentrations of D-AAs, together with D/L-AA ratios. This 2D-LC approach was later adapted to MS/MS detection and applied to plasma sample analysis [85]. Three-dimensional LC platforms have also been developed to achieve an even higher degree of selectivity. Compared to the 2D ones, these include a complementary separation dimension in between the reversed-phase (RP) and chiral ones, providing ultimate selectivity for the analysis of D-AAs. The main tradeoff in having the high degree of selectivity provided by the 2D- and 3D-LC methods is throughput, estimated to be lowest for the 3D approach (few samples/day) to low-medium for 2D-ones (~20–25 samples/day).

As higher throughput alternatives, two other promising LC-MS-based approaches have been published in recent years. The first [88] provides a fast and direct enantioseparation of 16 D-AAs, translating into much higher throughput (~90 samples/day). Moreover, this approach is more feasible to implement in different labs, since the availability of the needed instruments is relatively high. Still, the extent of the matrix effects and the influence of possible matrix-related interferences on method selectivity remain unknown, since they were not reported. The second approach [104] can offer baseline separation of (S)-NIFE derivatized D- and L-AAs in less than 20 min (~60 samples/day). The LC selectivity is complemented by the ion-mobility dimension that can give relevant information regarding the identity of the measured analytes, collisional cross-section being a supplementary annotation parameter. Still, the availability of ion-mobility mass spectrometers is not wide; nonetheless, depending on the available MS instruments, the LC separation could be readily adapted to different LC-MS platforms.

D-AA analysis by HPLC/LC-MS generally requires meticulous sample preparation (protein precipitation, phospholipid removal, pre-column derivatization), which is labor-intensive [104,105,106,107], though the actual separations can be fast (10–20 min via UHPLC or chiral crown-ether columns) [104], while multidimensional methods (2D/3D-HPLC) can extend to 45–120 min per sample [108]. Chiral derivatizing reagents (S-NIFE, FDLA) improve sensitivity and selectivity through enhanced ESI-MS ionization [34,104,108], enabling quantification limits that go as low as the femtomolar level and good precision (RSD < 10–15%) [104,109,110], but they can introduce risks such as unequal derivatization efficiency between enantiomers [105,108], artificial racemization during sample prep (especially under high-temperature or acidic/basic conditions) [108], and instability of certain derivatives (e.g., S-NIFE-D-histidine degrades within 24 h) [104]. Additionally, matrix effects from biological fluids (plasma, urine) can cause ion suppression or enhancement [104,105]. The large excess of L-AAs also poses interference risks [111], including false diastereomer formation from trace L-impurities in chiral reagents. Mitigating these challenges typically requires advanced multidimensional or ion-mobility separations, together with isotope-labeled internal standards [104,108].

These analytical constraints have contributed to a clear methodological distinction between discovery-grade profiling and routine clinical testing. Discovery-oriented 2D/3D-HPLC platforms prioritize comprehensive enantiomeric coverage and high resolving power through multidimensional chiral separation and extensive matrix cleanup, but require specialized instrumentation and longer analysis times. In contrast, routine clinical assays favor simplified UHPLC/LC-MS/MS or LC-IM-MS workflows enabling rapid, robust, and standardized quantification of targeted biomarkers such as D-Ser and D-Asn [104,107,108].

While chromatographic and MS-based methods remain the gold standard for accuracy and sensitivity, their complexity and cost have driven growing interest in biosensors. These provide a promising point-of-care alternative for D-AA evaluation in CKD, mainly using enzymatic (DAAO- or D-Ser dehydratase-based) electrochemical platforms [106], often enhanced with nanomaterials [112], offering rapid detection (seconds to under 2 min), high enantioselectivity without need for chiral separation, low detection limits, and portability suited for continuous monitoring [106,113]. Key limitations include enzyme instability under variable pH/temperature, matrix-related biofouling and interference, broad substrate specificity complicating single-analyte quantification, costly equipment for optical variants, and the ongoing need for calibration against reference chromatographic methods [113,114]. Further details on biosensor-based detection methods for D-AAs and their broader applications can be found in the dedicated review by Rosini et al. [113].

## 5. Inter-Organ Communication: The Gut–Kidney–Brain Axis

### 5.1. The Gut–Kidney Axis in CKD: Microbial D-Ser as a Renal Protective Signal

The gut microbiota is the primary exogenous source of D-Ser in humans [35]. While many bacteria produce D-AAs as components of their cell wall peptidoglycan, the specific species identified in feces include *Lactobacillus*, *Clostridium*, and *Ruminococcus*. In mice with AKI, these species increased while *Bifidobacterium* decreased, altering the D-Ser pool [96]. As previously described, microbially derived D-Ser is actively absorbed into the blood via the amino acid transporter ATB^0,+^ (SLC6A14), expressed in the intestinal epithelium [55]. Once in the systemic circulation, D-Ser is dominantly and quickly distributed to the kidney where it is filtered by the glomerulus and reabsorbed in the proximal tubules [70].

The protective and tissue-remodeling effects of D-Ser have been demonstrated predominantly in preclinical in vitro and animal models, while human evidence remains limited to observational studies, with no interventional clinical trials confirming its renoprotective efficacy. Nevertheless, the available preclinical data suggest that D-Ser exerts its renoprotective effects through multiple converging mechanisms. In hypoxia-stimulated tubular epithelial cells, it attenuates pro-inflammatory signaling by downregulating cytokines and chemokines including IL-6 and TNF-α [14,115]. Concurrently, D-Ser promotes tubular regeneration by stimulating the proliferation of Ki67-positive tubular cells, while mechanistically driving cell cycle re-entry for tissue remodeling via an mTOR/p18/Rheb-related pathway [31]. Additionally, it suppresses hypoxia-induced tubular damage and prevents mitochondrial fragmentation, indicating a role in preserving mitochondrial integrity under ischemic conditions [14].

A question therefore arises: if D-Ser exerts renoprotective effects, why does CKD continue to progress despite increasing circulating D-Ser levels as eGFR declines? This apparent paradox reflects the context- and exposure-dependent biology of D-Ser. At low-dose exposure in experimental models, D-Ser can promote compensatory tubular remodeling and proliferation through mTOR-related signaling [18,31]. As CKD progresses, impaired renal clearance increases circulating D-Ser [86,89], while the underlying disease continues to cause nephron loss, tubular hypoxia, inflammation, and fibrosis. In addition, sustained or excessive D-Ser exposure may become maladaptive through NMDA-receptor/DAAO-associated oxidative stress and cellular stress responses linked to tubular senescence, apoptosis, and pro-fibrotic signaling [17,33,82]. Thus, rising circulating D-Ser should be interpreted primarily as a clearance-sensitive biomarker that may also contribute to injury under some conditions, not as evidence that endogenous D-Ser is sufficient to arrest CKD [17,86]. The “double-edged sword” model is a useful working hypothesis, but its concentration thresholds and relevance to human CKD remain to be established [26].

Experimental models collectively support a causal renoprotective role for microbially derived D-Ser. In ischemia–reperfusion AKI models, germ-free mice exhibited worsened histological kidney damage relative to conventionally colonized controls. This injury pattern was rescued by fecal microbiota transplantation from normal mice, directly implicating the gut microbiota as a source of renoprotective signals. Oral D-Ser supplementation at 20 mM recapitulated this protection, significantly mitigating kidney injury, reducing F4/80-positive immune cell infiltration, and decreasing albuminuria [96]. In a 5/6 nephrectomy CKD model, long-term supplementation at the same dose improved survival and preserved renal function, evidenced by reductions in blood urea nitrogen and serum creatinine [115]. Furthermore, low-dose D-Ser promoted compensatory renal hypertrophy in uninephrectomized mice without adverse effects, suggesting a role in adaptive remodeling beyond acute injury [31]. Taken together, these findings indicate that D-Ser mediates gut–kidney crosstalk not only in the context of acute ischemic insult, but also in chronic nephron loss and physiological adaptation.

The gut–kidney relationship is bidirectional (Figure 2). Renal injury itself rapidly disrupts gut microbial richness and community structure (dysbiosis), reducing D-Ser availability [96]. In CKD, this dynamic is further compounded by urea accumulation: urea diffuses into the intestinal lumen, where bacterial urease converts it to ammonia, elevating luminal pH and promoting the production of uremic toxins such as indoxyl sulfate and p-cresyl sulfate [48,116]. This altered environment selectively favors proteolytic, toxin-producing bacteria at the expense of beneficial D-Ser-producing species [47,48]. The resulting depletion of microbial D-Ser withdraws a key renoprotective signal (reduced mTOR/p18 activation), rendering the kidney increasingly vulnerable to further injury and disease progression, thereby establishing a self-reinforcing cycle in which renal dysfunction and gut dysbiosis perpetuate one another [96].

Given this bidirectional vulnerability, restoring microbial D-Ser availability has emerged as a therapeutic strategy. Direct oral supplementation with D-Ser or D-Ala has been proposed as a dietary and pharmacological intervention in CKD [115]. Probiotic approaches, including *Lactobacillus*-based formulations, are being explored to restore microbial equilibrium and, by extension, D-Ser availability [49,116]. Dietary interventions such as sake lees, which combine fiber, live bacteria, and D-Ser, are currently being evaluated in clinical trials for their capacity to reduce uremic toxin burden [50,117]. Additionally, risperidone, a DAAO inhibitor, represents a pharmacological strategy to enhance endogenous D-Ser bioavailability by attenuating its enzymatic degradation [115], as tested in patients with schizophrenia [118].

The translation of D-Ser into a therapeutic strategy faces several unresolved challenges. These include the predominantly preclinical nature of current evidence, small and observational human datasets, and uncertainties regarding its pharmacokinetics, tissue distribution, and therapeutic window [31,53]. A major translational challenge is the apparent context- and exposure-dependent behavior of D-Ser: low-dose exposure promotes compensatory tubular remodeling and cell proliferation [31], whereas sustained or higher exposure has been associated with tubular injury, senescence, apoptosis, and NMDA receptor/NOX4-associated oxidative damage [32,33]. Moreover, circulating D-Ser levels increase as GFR declines, making it difficult to distinguish an active adaptive response from a consequence of impaired renal clearance [18]. The limited evidence for sex-dependent responses, together with differences between microbiota-derived and pharmacologically administered D-Ser, further complicates clinical translation [96]. Because D-Ser cannot independently overcome the multifactorial drivers of CKD progression and human interventional trials demonstrating sustained renal benefit are lacking [53,115], it is currently best regarded as a clearance-sensitive biomarker and context-dependent modulator of renal homeostasis rather than an established therapeutic strategy.

### 5.2. The Kidney–Brain Axis in CKD: D-AAs as Drivers of Neurological Injury

The kidney–brain axis represents a complex bidirectional communication network where structural, hemodynamic, and chemical signaling links the two organs [119,120], ensuring that dysfunction in one system triggers cascading pathological effects in the other [116]. Cognitive impairment emerges early in CKD and scales with nephron loss. In a cross-sectional study, mild cognitive impairment occurred significantly more frequently in patients with eGFR < 45 mL/min/1.73 m^2^ (OR = 2.35) compared to those with eGFR > 60, establishing GFR decline as an independent neurological risk factor [121].

The translocation of D-AAs into the central nervous system is mediated by specialized facilitative solute carriers at the blood–brain barrier (BBB), including LAT1 (SLC7A5), ASCT1 (SLC1A4), and ASCT2 (SLC1A5) [24,62]. This axis is further destabilized by the gut-derived uremic toxin indoxyl sulfate, which activates the aryl hydrocarbon receptor (AhR) in cerebral endothelial cells [116,120]. Activation of the AhR pathway triggers the downregulation of critical tight junction proteins [122], specifically occludin and zonula occludens-1, thereby increasing BBB permeability and allowing a massive influx of neurotoxic metabolites and systemic pro-inflammatory cytokines (IL–1β, TNF–α) into the brain parenchyma [120,123].

Once in the central nervous system, pathologically elevated D-Ser and D-Ala disrupt synaptic homeostasis by functioning as potent endogenous co-agonists at the GluN1 subunit (glycine site) of the NMDA receptor [26]. While physiological levels of these molecules are essential for memory, uremic accumulation leads to NMDA receptor overstimulation, triggering excessive cellular calcium influx and excitotoxic cell death [106]. This pathway is amplified by D-Asp, which acts as an agonist at the GluN2 glutamate-binding pocket [26,124], and by reactive astrocytes that release excessive D-Ser to preferentially activate extrasynaptic NMDA receptors, a mechanism directly linked to neuronal apoptosis, dendritic spine loss, and synaptic pruning [24].

Clinical evidence validates this molecular bridge, demonstrating that the altered chiral profile of circulating amino acids correlates with global cerebral dysfunction [50,82]. Specifically, increased plasma D-Ser and intracranial D-Asn levels are strongly associated with impairments in executive function and memory processing speed [119]. These metabolic imbalances accelerate the progression of uremic encephalopathy, Alzheimer’s disease, and cerebral atrophy as renal function declines [123], positioning D-AAs as potential molecular contributors to kidney–brain communication [18,50].

## 6. Conclusions and Future Directions

This review highlights the growing recognition of D-AAs as biologically active molecules rather than simple metabolic by-products. Current evidence indicates that they act as sensitive indicators of nephron integrity, regulators of inter-organ communication, and promising therapeutic candidates in CKD. Among them, plasma D-Ser and D-Asn have shown promising diagnostic performance in comparison with conventional renal biomarkers. Moreover, their FE profiles offer additional insights into tubular reabsorptive capacity, providing functional information that cannot be obtained through single-marker assessments alone. Within the gut–kidney–brain axis, D-AAs emerge as key molecular links connecting microbial metabolism, renal homeostasis, and neurological dysfunction, thereby offering an integrated framework for understanding the multisystem manifestations of CKD.

Although D-Ser shows a strong correlation with measured GFR, most of the evidence comes from small, cross-sectional studies and a limited number of research groups. Large prospective investigations involving diverse, multi-ethnic populations are therefore needed to validate these findings, define robust reference ranges, and assess predictive performance across CKD stages [35,53,63]. In addition, mainstream approaches (LC–MS/MS, chiral HPLC) lack unified protocols, contributing to interlaboratory variability and inconsistent results. This hinders clinical translation of D-AAs, making harmonized protocols, validated reference materials, and ring trials essential for reliable implementation [26,35,62]. Sex differences in renal amino acid metabolism are also still not sufficiently considered in current studies. Because sex hormones modulate transporter expression and tubular handling, failure to stratify by sex may confound associations between D-AAs and disease outcomes. Future research should therefore systematically include sex-stratified analyses to support more precise, personalized approaches in nephrology [53,63]. Finally, current knowledge relies mainly on static metabolite measurements, which cannot resolve dynamic processes such as synthesis, degradation, transport, or inter-organ exchange. To better understand these processes, future work will need to combine isotopic flux analysis with multi-omics approaches, allowing a more complete and dynamic view of renal D-AA handling in both health and disease [53,63]. Future validation should therefore extend beyond analytical performance to assess calibration, reclassification, reproducibility, cost, and the ability of D-AA biomarkers to improve clinical outcome prediction.

Beyond biomarker validation, the development of suitable monitoring technologies represents another important translational challenge. Compared with gas-phase breath analysis, sweat and exhaled breath condensate (EBC) may provide more plausible matrices for monitoring D-AAs, as these nonvolatile molecules would not be expected to partition into the gas phase in the same way as ammonia or volatile organic compounds [125]. Wearable and microfluidic platforms have demonstrated the feasibility of non-invasively measuring urea and creatinine in sweat [126,127], showing that non-invasive biofluid sensing and wireless readout are feasible for renal analytes. However, whether D-Ser, D-Asn, or their D/L ratios can be reliably detected in these matrices and reflect plasma or urinary levels remains unknown. Factors such as breathing pattern, sweat rate, and oral contamination would also require careful characterization, making this a promising but currently unvalidated direction for future research.

## Figures and Tables

**Figure 1:**
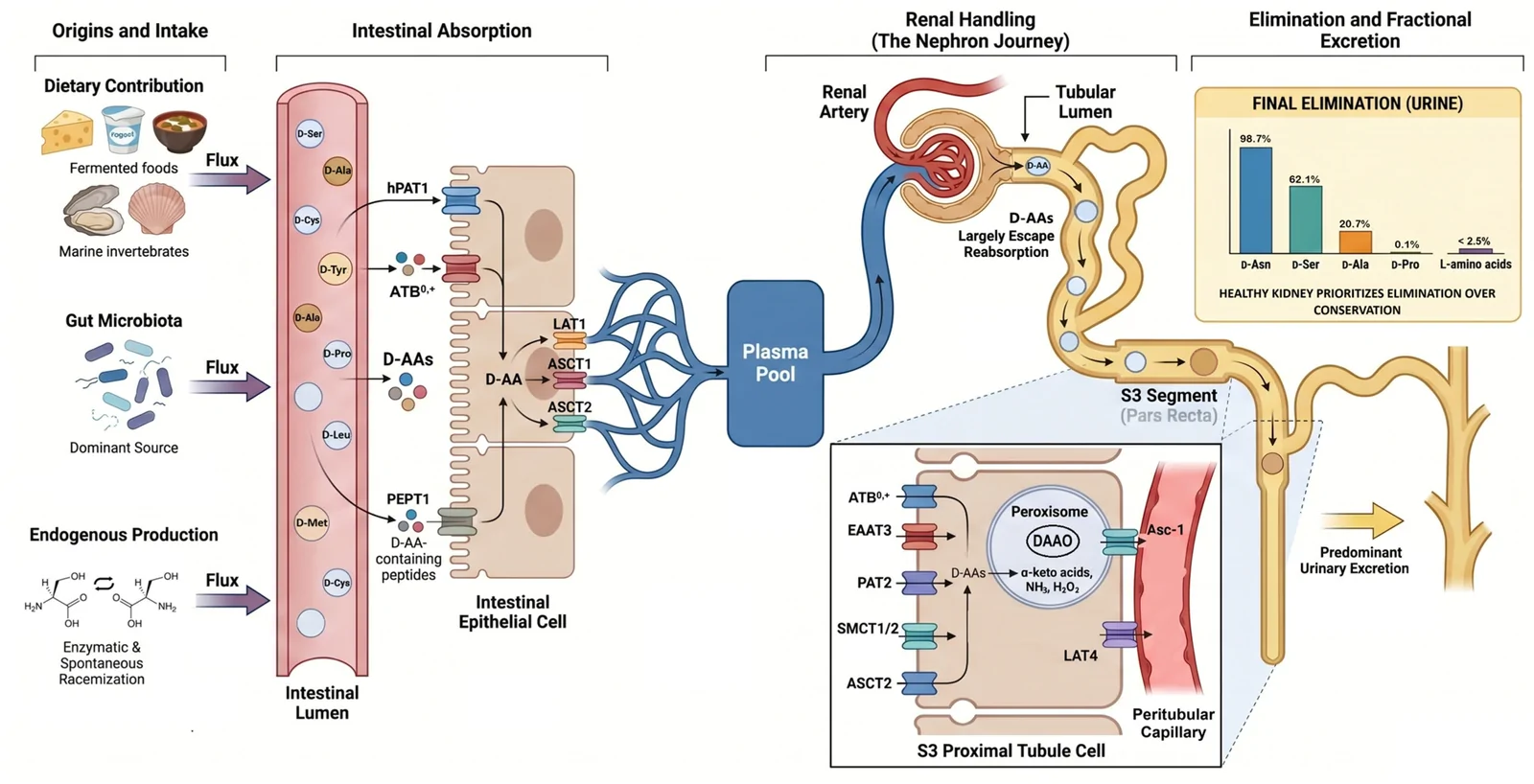
Physiological trajectory and renal handling of D-AAs, from dietary, microbial, and endogenous origins through intestinal absorption, glomerular filtration, and stereoselective proximal tubule handling (apical uptake, DAAO-mediated degradation, basolateral efflux) to urinary excretion. DAAO: D-amino acid oxidase; S3: proximal tubule segment 3 (pars recta). Conceptual framework developed by the authors and visually refined with the assistance of NotebookLM (Google).

**Figure 2:**
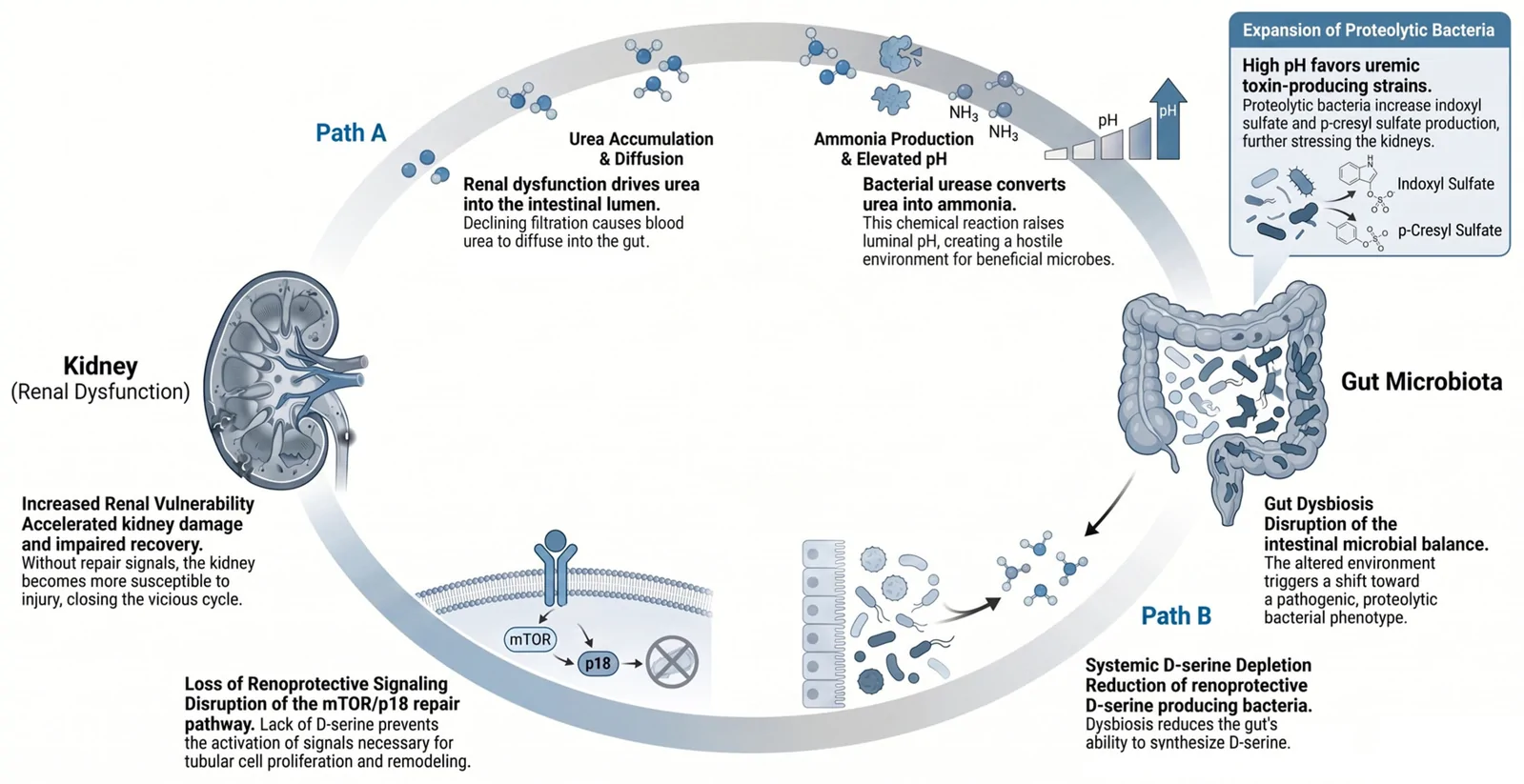
Bidirectional gut–kidney axis in CKD, as supported by preclinical evidence. Path A: Renal dysfunction drives intestinal dysbiosis via urea accumulation and ammonia-mediated pH elevation, favoring proteolytic bacteria and uremic toxin production. Path B: Dysbiosis depletes microbial D-serine, disrupting mTOR/p18-dependent tubular repair signaling and increasing renal vulnerability. Conceptual framework developed by the authors and visually refined with the assistance of NotebookLM (Google).

**Table 1:** Comparison of selected emerging biomarkers in CKD, highlighting the translational potential of D-AAs.

Biomarker	Biomarker Class	Primary Clinical Application	Key Advantage	Main Limitation	Clinical Validation Level	Ref.
KIM-1 (Kidney Injury Molecule-1)	Tubular injury protein (glycoprotein)	Early detection of proximal tubular damage; prediction of CKD progression	Rapidly shed during active injury; near absent in healthy kidneys	Lack of standardized clinical cutoffs; levels may decline in very advanced CKD due to loss of functional tubular mass	Clinical validation (widely used in nephrotoxicity and cohort studies)	[75]
NGAL (Neutrophil Gelatinase-Associated Lipocalin)	Tubular injury/inflammation protein	Early detection of active tubular stress/injury; predicting cardiorenal risk	Rapidly upregulated and excreted in urine/blood within hours of injury, well before GFR declines	Confounded by general systemic inflammation, infections, and sepsis (acute-phase reactant)	Clinical implementation (widely validated for CKD progression)	[76]
Uromodulin (Tamm–Horsfall Protein)	Loop of Henle glycoprotein/nephron mass marker	GFR-independent index of tubular mass; predicts CKD onset, ESKD and CV risk	Exclusive to the thick ascending limb; reflects parenchymal integrity and anti-inflammatory function	Sensitive to urine storage conditions; influenced by uromodulin gene variants and sex hormones	Clinical validation (extensively evaluated in large population studies)	[77]
suPAR (Soluble Urokinase Receptor)	Systemic inflammation/podocyte stress	Predicting CKD onset, eGFR decline, and cardiorenal events	Predicts GFR decline despite normal baseline function; directly mediates podocyte injury	High analytical variability across assays; heavily influenced by general chronic inflammatory states	Clinical validation (substantial cohort data including CRIC and CKiD)	[78]
FGF-23 (Fibroblast Growth Factor 23)	Mineral metabolism hormone	Predicting CKD-MBD, rapid GFR decline, and cardiovascular risk	Rises very early in CKD, well before alterations in serum phosphate or parathyroid hormone are detectable	Influenced by dietary phosphate intake; marked assay variability (intact vs. C-terminal)	Clinical validation (used in clinical research and risk assessment models)	[79,80]
SDMA (Symmetric Dimethylarginine)	Methylated arginine derivative	Early CKD diagnosis & long-term mortality risk assessment	Freely filtered; >90% renally excreted and unaffected by diet, muscle mass, inflammation, obesity, or diabetes	Lacks absolute organ specificity; exhibits mild pro-inflammatory biological activity (activates NF-κB and ROS)	Clinical validation (validated in clinical trials, large-scale meta-analyses, and animal models)	[81]
D-Ser, D-Asn	Chiral metabolite (D-AA)	eGFR measurement; early CKD prognosis and urine volume estimation (see Table 2)	Highly correlated with measured GFR (inulin clearance); less dependent on body composition	Require enantioselective assays (2D/3D-HPLC) for blood/urine isolation; D-Ser is influenced by neurological conditions and diet	Clinical validation (validated in living donor and kidney transplant recipient cohorts against measured GFR)	[20,21,64]

**Table 3:** Analytical methods used to generate D-AA data related to CKD.

Nr.	Platform	Chiral Principle	Detection	Sample Type	Clinical Outputs	Expected Throughput	Instrument Availability	Ref.
1	2D-HPLC–FL	Direct—reversed-phase 1st dimension × chiral 2nd dimension	Fluorescence (NBD-F derivatives)	Plasma, serum, urine	Absolute conc., D/L ratio	Low-moderate	Low—few specialist labs	[19,20,21,22,23,69,82,83,89]
2	3D-HPLC–FL	Direct—reversed-phase × ion-exchange × chiral	Fluorescence (NBD-F derivatives)	Plasma	Absolute conc., D/L ratio	Low	Very low—specialized custom three-column setup	[84,103]
3	2D-LC–MS/MS	Direct—reversed-phase 1st dimension × chiral 2nd dimension + MS	Tandem MS (MRM); NBD-F aids ionization	Plasma	Absolute conc., D/L ratio	Low–moderate	Moderate—triple-quads are common, but the 2D-LC front end is not	[85]
4	Chiral-column LC–MS/MS	Direct—chiral stationary phase	Tandem MS (MRM)	Urine (IgA nephropathy)	Absolute conc.	High (tens; ~15 min/run)	High—standard LC–MS/MS + a commercial chiral column	[88]
5	Chiral-derivatization LC–IM–MS	Indirect—(S)-NIFE chiral derivatization, RP separation	DIA-MS + ion mobility (CCS)	Plasma (CKD stages 4–5)	Relative abundance/Absolute conc.	Moderate–High (tens; 20 min/run)	Moderate–High—commercial reagent + standard RP; IM-MS increasingly available	[104]

## Data Availability

No new data were created or analyzed in this study. Data sharing is not applicable to this article.

## Abbreviations

The following abbreviations are used in this manuscript:

2D-HPLC/2D-LC	Two-dimensional (high-performance) liquid chromatography
3D-HPLC/3D-LC	Three-dimensional (high-performance) liquid chromatography
AhR	Aryl hydrocarbon receptor
AKI	Acute kidney injury
Ala	Alanine
ALS	Amyotrophic lateral sclerosis
Asc-1 (SLC7A10)	Alanine-serine-cysteine antiporter 1
ASCT1 (SLC1A4)	Alanine-serine-cysteine transporter 1
ASCT2 (SLC1A5)	Alanine-serine-cysteine transporter 2
Asn	Asparagine
Asp	Aspartate/aspartic acid
ATB0,+ (SLC6A14)	Amino acid transporter B0,+
AUC	Area under the curve
B0AT1 (SLC6A19)	Neutral amino acid transporter B0AT1
BBB	Blood–brain barrier
CCS	Collisional cross-section
CKD	Chronic kidney disease
CKD-MBD	Chronic kidney disease–mineral and bone disorder
CKiD	Chronic Kidney Disease in Children (study cohort)
CRIC	Chronic Renal Insufficiency Cohort (study)
Cys	Cysteine
CysC	Cystatin C
D-AA(s)	D-amino acid(s)
DAAO	D-amino acid oxidase
DIA	Data-independent acquisition
D/L (ratio)	Ratio of D- to L-enantiomer concentration
DKD	Diabetic kidney disease
EAAT3 (SLC1A1)	Excitatory amino acid transporter 3
EBC	Exhaled breath condensate
eGFR	Estimated glomerular filtration rate
ESI-MS	Electrospray ionization mass spectrometry
ESKD	End-stage kidney disease
FDLA	1-Fluoro-2,4-dinitrophenyl-5-leucinamide
FE	Fractional excretion
FGF-23	Fibroblast growth factor 23
GFR	Glomerular filtration rate
Glu	Glutamate/glutamic acid
H_2_O_2_	Hydrogen peroxide
hPAT1 (SLC36A1)	Human proton-coupled amino acid transporter 1
HPLC	High-performance liquid chromatography
IgAN	Immunoglobulin A nephropathy
IL-6	Interleukin 6
IM-MS	Ion mobility–mass spectrometry
KIM-1	Kidney injury molecule-1
LAT1 (SLC7A5)	L-type amino acid transporter 1
LAT4 (SLC43A2)	L-type amino acid transporter 4
LC–MS/MS	Liquid chromatography–tandem mass spectrometry
Leu	Leucine
Lys	Lysine
Met	Methionine
MRM	Multiple reaction monitoring
MS	Mass spectrometry
mTOR	Mammalian target of rapamycin
NBD-F	4-Fluoro-7-nitrobenzofurazan
NF-κB	Nuclear factor kappa-light-chain-enhancer of activated B cells
NGAL	Neutrophil gelatinase-associated lipocalin
NH_3_	Ammonia
(S)-NIFE	(S)-N-(4-nitrophenoxycarbonyl) phenylalanine 2-methoxyethyl ester
NMDA	N-methyl-D-aspartate
NOX4	NADPH oxidase 4
PAT2 (SLC36A2)	Proton-coupled amino acid transporter 2
PEPT1 (SLC15A1)	Peptide transporter 1
Phe	Phenylalanine
Pro	Proline
rBAT (SLC3A1)	Related to b0,+ amino acid transporter
ROS	Reactive oxygen species
RP	Reversed phase (chromatography)
sCr	Serum creatinine
SDMA	Symmetric dimethylarginine
Ser	Serine
SLC	Solute carrier (transporter gene family)
SMCT1/SMCT2 (SLC5A8/SLC5A12)	Sodium-coupled monocarboxylate transporter 1/2
suPAR	Soluble urokinase plasminogen activator receptor
Thr	Threonine
TNF-α	Tumor necrosis factor alpha
Trp	Tryptophan
Tyr	Tyrosine
UHPLC	Ultra-high-performance liquid chromatography
Val	Valine
